# Reproducibility of respiratory mechanics measurements in patients on invasive mechanical ventilation

**DOI:** 10.5935/0103-507X.20200068

**Published:** 2020

**Authors:** José da Natividade Menezes Júnior, Ludmilla Mota Silva, Leonardo José Morais Santos, Helena França Correia, Wende Lopes, Virgínia Eugênia Pinheiro e Silva, Jorge Luis Motta dos Anjos, Bruno Prata Martinez

**Affiliations:** 1 Hospital Geral Roberto Santos - Salvador (BA), Brazil.; 2 Postgraduate Program in Interactive Processes of Organs and Systems, Universidade Federal da Bahia - Salvador (BA), Brazil.; 3 Universidade do Estado da Bahia - Salvador (BA), Brazil.; 4 Hospital Universitário Professor Edgard Santos - Salvador (BA), Brazil.; 5 Postgraduate Program in Medicine and Health, Universidade Federal da Bahia - Salvador (BA), Brazil.

**Keywords:** Respiratory mechanics, Respiration, artificial, Reproducibility of results, Mecânica respiratória, Respiração artificial, Reprodutibilidade dos testes

## Abstract

**Objective:**

To evaluate the intra- and interexaminer reproducibility of measurements of the resistance and static and dynamic compliance of the respiratory system in patients on mechanical ventilation.

**Methods:**

This was an analytical study conducted with individuals aged ≥ 18 years who were on invasive mechanical ventilation and had no clinical diagnosis of respiratory system disease and/or chest abnormality. Three measurements of respiratory mechanics were performed with a 1-minute interval between them. The first and third measurements were performed by examiner A, the second by examiner B. The values for the resistance and static and dynamic compliance of the respiratory system were compared using the intraclass correlation coefficient.

**Results:**

A total of 198 measurements of respiratory mechanics were performed for 66 patients on mechanical ventilation. The patients had a mean age of 52.6 ± 18.6 years and a mean body mass index of 21.6 ± 2.1kg/m^2^; a surgical profile (61.5%) and female sex (53.8%) were predominant. Mean values were obtained for the three measurements of respiratory system resistance (A1: 15.7 ± 6.8cmH_2_O/L/s; B1: 15.7 ± 6.4cmH_2_O/L/s and A2: 15.9 ± 6.2cmH_2_O/L/s), respiratory system static compliance (A1: 42.1 ± 13.7mL/cmH_2_O; B1: 42.4 ± 14.6mL/cmH_2_O and A2: 42.2 ± 14.5mL/cmH_2_O) and respiratory system dynamic compliance (A1: 21.3 ± 7.3mL/cmH_2_O; B1: 21.4 ± 7.5mL/cmH_2_O and A2: 21.3 ± 6.2mL/cmH_2_O). The intraclass correlation coefficient was also calculated for respiratory system resistance (R = 0.882 and p = 0.001; R = 0.949 and p = 0.001 - interexaminer A1 *versus* B and B *versus* A2, respectively; R = 0.932 and p = 0.001 - intraexaminer); respiratory system static compliance (R = 0.951 and p = 0.001; R = 0.958 and p = 0.001 - interexaminer A1 *versus* B and B *versus* A2, respectively; R = 0.965 and p = 0.001 - intraexaminer) and respiratory system dynamic compliance (R = 0.957 and p = 0.001; R = 0.946 and p = 0.001 - interexaminer A1 *versus* B and B *versus* A2, respectively; R = 0.926 and p = 0.001 - intraexaminer).

**Conclusion:**

The measurements of resistance and static and dynamic compliance of the respiratory system show good intra- and interexaminer reproducibility for ventilated patients.

## Abstract (pt)

**Objetivo:**

Avaliar a reprodutibilidade intra e interexaminador das mensurações da resistência e das complacências estática e dinâmica do sistema respiratório em pacientes sob ventilação mecânica.

**Métodos:**

Trata-se de estudo analítico realizado com indivíduos com idade ≥ 18 anos, em ventilação mecânica invasiva, que não tinham diagnóstico clínico de doença do aparelho respiratório e/ou anormalidade de caixa torácica. Foram realizadas três aferições da mecânica respiratória com intervalo de 1 minuto entre elas. A primeira e a terceira aferições foram realizadas pelo avaliador A e a segunda aferição, pelo avaliador B. A comparação dos valores de resistência e complacências estática e dinâmica do sistema respiratório foi calculada por meio do coeficiente de correlação intraclasse.

**Resultados:**

Foram realizadas 198 aferições da mecânica respiratória em 66 pacientes sob ventilação mecânica, com idade média de 52,6 ± 18,6 anos, índice de massa corporal médio de 21,6 ± 2,1kg/m^2^, predomínio do perfil cirúrgico (61,5%) e sexo feminino (53,8%). Foram obtidos valores médios das três aferições para resistência do sistema respiratório (A1: 15,7 ± 6,8cmH_2_O/L/s; B1: 15,7 ± 6,4cmH_2_O/L/s e A2: 15,9 ± 6,2cmH_2_O/L/s), para complacência estática do sistema respiratório (A1: 42,1 ± 13,7mL/cmH_2_O; B1: 42,4 ± 14,6mL/cmH_2_O e A2: 42,2 ± 14,5mL/cmH_2_O) e para complacência dinâmica do sistema respiratório (A1: 21,3 ± 7,3mL/cmH_2_O; B1: 21,4 ± 7,5mL/cmH_2_O e A2: 21,3 ± 6,2mL/cmH_2_O). Também foram encontrados valores do coeficiente de correlação intraclasse para resistência do sistema respiratório (R = 0,882 e p = 0,001; R = 0,949 e p = 0,001 - interexaminadores A1 versus B e B versus A2, respectivamente; R = 0,932 e p = 0,001 - intraexaminador); complacência estática do sistema respiratório (R = 0,951 e p = 0,001; R = 0,958 e p = 0,001 - interexaminadores A1 versus B e B versus A2, respectivamente; R = 0,965 e p = 0,001 - intraexaminador) e complacência dinâmica do sistema respiratório (R = 0,957 e p = 0,001; R = 0,946 e p = 0,001 - interexaminadores A1 versus B e B versus A2 respectivamente; R = 0,926 e p = 0,001 - intraexaminador).

**Conclusão:**

A mensuração de mecânica respiratória apresenta boa reprodutibilidade intra e interexaminador para as aferições de resistência e complacências estática e dinâmica do sistema respiratório em pacientes ventilados.

## INTRODUCTION

Mechanical ventilation (MV) is frequently used in the care of critically ill patients to promote rest for the respiratory muscles and allow adequate tissue oxygen supply. Although it is essential for survival, this type of support is not without risks, and attention should be paid to the monitoring of respiratory mechanics parameters as changes in these parameters may increase the risk of MV-induced lung injury. Currently, there is growing concern regarding MV-induced lung injury caused by mechanical stress on the lung parenchyma, which can have consequences at both the pulmonary and systemic levels.^(1,2)^

An understanding of respiratory mechanics allows the use of the parameters evaluated as guidelines for adjusting MV settings to reduce associated injury^(1)^ and assists in determining the indications for and evaluating the results of physical therapy interventions.^(2)^

Ventilator-induced lung injury is a form of iatrogenic injury caused by inadequate maintenance of ventilation, especially in patients with impaired respiratory mechanics. This process leads to the release of inflammatory mediators and perpetuates dependence on ventilatory support.^(3)^ To minimize these risks, ventilation strategies were developed to prevent lung injury. Monitoring and analysis of the respiratory system provide support for understanding ventilatory dynamics and thus optimizing ventilatory support.^(4,5)^

Pulmonary mechanics is the study of the forces that act on the respiratory system. The measures used in this study are compliance and resistance.^(6)^ Compliance is associated with pulmonary distensibility and is equivalent to the volume variation divided by the pressure variation. Resistance is related to air conduction and is influenced by factors such as the presence of secretions in the airways and narrowing of the airways. The monitoring of these variables allows the longitudinal comparison of data over the period during which the patient remains on MV.^(1,6,7)^ To allow such comparisons, there must be good reproducibility among examiners to confer clinical significance for the management of critically ill patients.

Although respiratory mechanics measurements are a relevant parameter for monitoring the impedance of the respiratory system, there are no studies that describe the reproducibility of the relevant measurements: airway resistance (Raw) and static (Cst rs) and dynamic (Cdyn rs) compliance of the respiratory system. Thus, this study evaluated the intra- and interexaminer reproducibility of the Raw, Cst rs and Cdyn rs measurements in patients on MV.

## METHODS

This is an analytical study conducted in the intensive care units (ICUs) of a large public hospital in the city of Salvador, Bahia, Brazil. Participants aged ≥ 18 years were included if they were on invasive MV in assisted-controlled modes; were sedated and did not require interaction with the mechanical ventilator (i.e., they were fully adapted to the ventilator), as visualized by graphical analysis of the flow *versus* time and pressure *versus* time curves; had stable hemodynamics, characterized by the nonuse of vasoactive or inotropic drugs or the use of low doses (up to 0.3mg/kg); did not have fractures (rib cage, spine or hip); and had no clinical diagnosis of respiratory system disease and/or chest abnormalities. Patients with a mean blood pressure change greater than 20% compared to baseline, systolic blood pressure (SBP) < 90mmHg visualized by invasive blood pressure measurement and a drop in oxygen saturation (SpO_2_) < 90% during the measurements were excluded.

The study was approved by the Research Ethics Committee of *Hospital Geral Roberto Santos* under CAAE no. 57895516.8.1001.5028. After the informed consent form was signed by a family member and/or guardian, three measurements of respiratory mechanics were performed by two examiners, with a 1-minute interval between measurements. The first and third measurements were performed by examiner A and the second by examiner B.

Respiratory system mechanics were evaluated using flow interruption at the end of inspiration, which required the use of the volume mode of ventilation and an inspiratory pause time of 0.5 seconds.^(7)^ The values used for these measurements were a tidal volume (VT) of 6mL/kg of ideal weight, a flow of 40 - 60L/minute, approximately 10% of VT and a respiratory rate of 15 breaths per minute.^(7)^

The Cst rs value was obtained using the formula $\left(\mathrm{Cst}\;\mathrm{rs}\right)\;=\;\mathrm{VT}/\mathrm{Pplateau}$ -positive end-expiratory pressure (PEEP); for Cdyn rs, the formula $\left(\mathrm{Cdyn}\;\mathrm{rs}\right)=\mathrm{VT}/\mathrm{Ppeak}$ - PEEP was used. The Raw values were obtained using the formula $\mathrm{Raw}=\left(\mathrm{Ppeak}-\mathrm{Pplateau}\right)/\mathrm{flow}\left(L\right)$.

The comorbidities described in table 1 refer to hypertension, diabetes, chronic renal failure, dyslipidemia, acquired immunodeficiency syndrome, previous stroke, atrial fibrillation and chronic obstructive arterial disease.

**Table 1:** Clinical and demographic data of the included patients

Variable	n (%)	Mean ± SD
Age		52.6 ± 18.6
BMI (kg/m^2^)		21.6 ± 2.1
Sex		
Male	31 (46.2)	
Female	35 (53.8)	
Admission profile		
Clinical	27 (40.9)	
Surgical	39 (59.1)	
Reason for ICU admission		
Postoperative neurological surgery	27 (40.9)	
Neurological disease	18 (27.3)	
Postoperative abdominal surgery	8 (12.1)	
Sepsis	5 (7.5)	
Postoperative vascular surgery	3 (4.5)	
Renal failure	2 (3)	
Hepatic failure	1 (1.5)	
Cancer	1 (1.5)	
Postpartum complications	1 (1.5)	
Presence of comorbidities	33 (50)	

SD - standard deviation; BMI - body mass index; ICU - intensive care unit.

The Statistical Package for Social Sciences (SPSS) version 22.0 for Windows was used for data tabulation and analysis. Data normality was determined using the Kolmogorov-Smirnov test. The Raw, Cst rs and Cdyn rs values of the three measurements were compared using the intraclass correlation coefficient (ICC), with a significance level of p < 0.05.

The ICC is used to determine the reliability of measurements. The closer the ICC is to 1, the greater the correlation.^(8)^ Measurements can be classified as having reasonable reproducibility if the ICC is between 0.4 and 0.59, good reproducibility if it is between 0.6 and 0.74 and excellent reproducibility if it is above 0.74.^(9)^

## RESULTS

**Figure 1:**
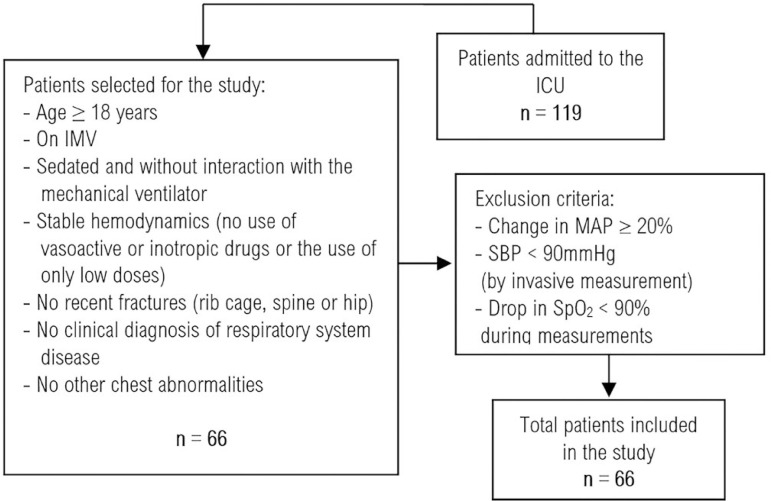
Flowchart of patient recruitment to the study. ICU - intensive care unit; IMV - invasive mechanical ventilation; MAP - mean arterial pressure; SBP - systolic blood pressure; SpO_2_ - oxygen saturation.

A total of 198 respiratory mechanics measurements were performed for 66 patients on MV included in the study (Figure 1). Of these, 53.8% were female, with a mean age of 52.6 ± 18.6 years, a mean body mass index (BMI) of 21.6 ± 2.1kg/m^2^, and a predominance of the surgical profile (59, 1%) and of neurological surgeries, as described in table 1.

**Figure 2:**
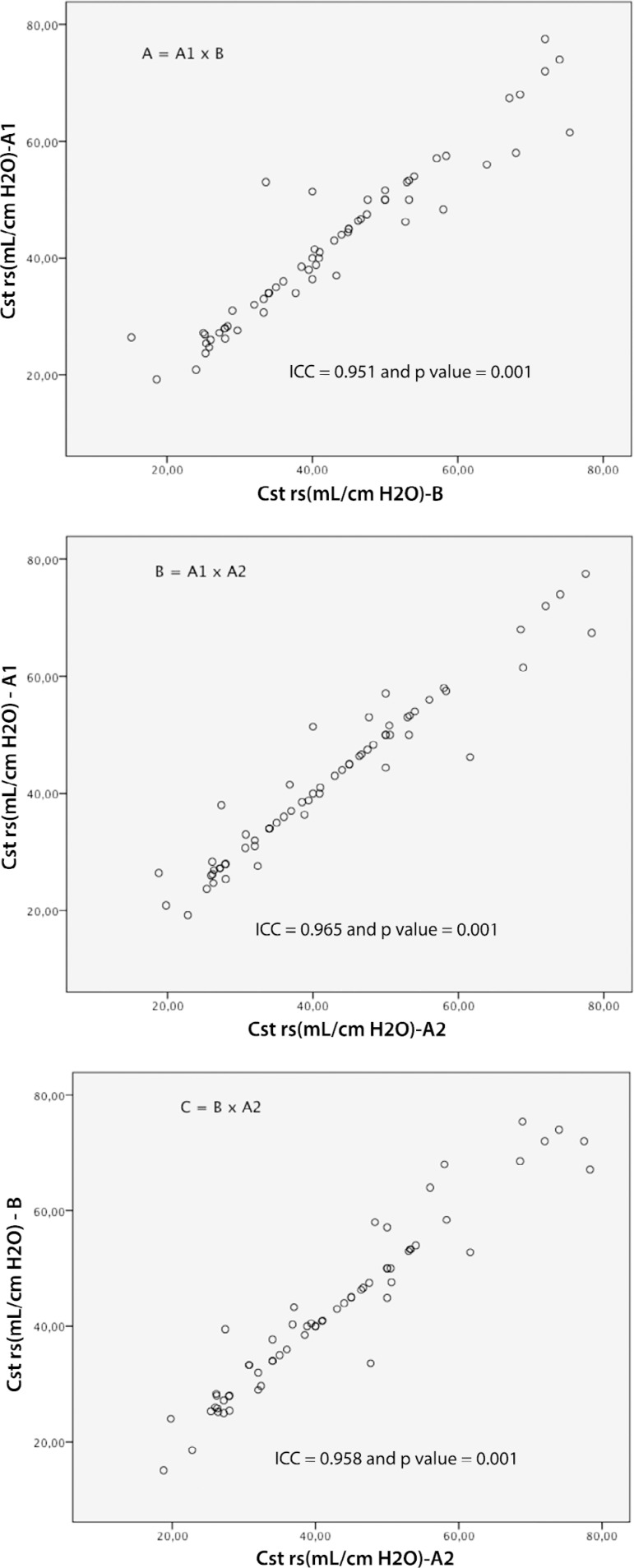
Analysis of the intraclass correlation coefficients for the measurements of respiratory system static compliance, interexaminer (A = A1 x B and C = B x A2) and intraexaminer (B = A1 x A2), with n = 66. ICC - intraclass correlation coefficient; Cst rs - static compliance of the respiratory system.

**Figure 3:**
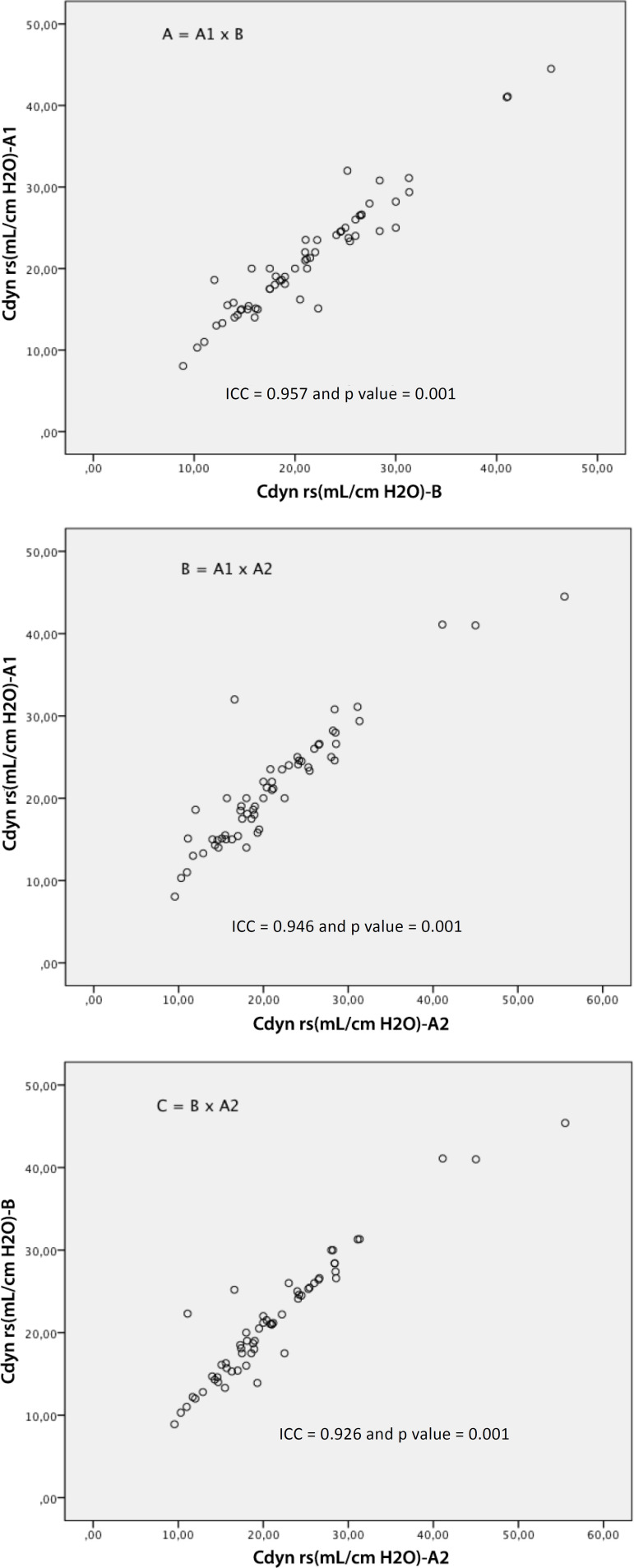
Analysis of the intraclass correlation coefficients for the measurements of respiratory system dynamic compliance, interexaminer (A = A1 x B and C = B x A2) and the intraexaminer (B = A1 x A2). ICC - intraclass correlation coefficient; Cdyn rs - dynamic compliance of the respiratory system.

**Figure 4:**
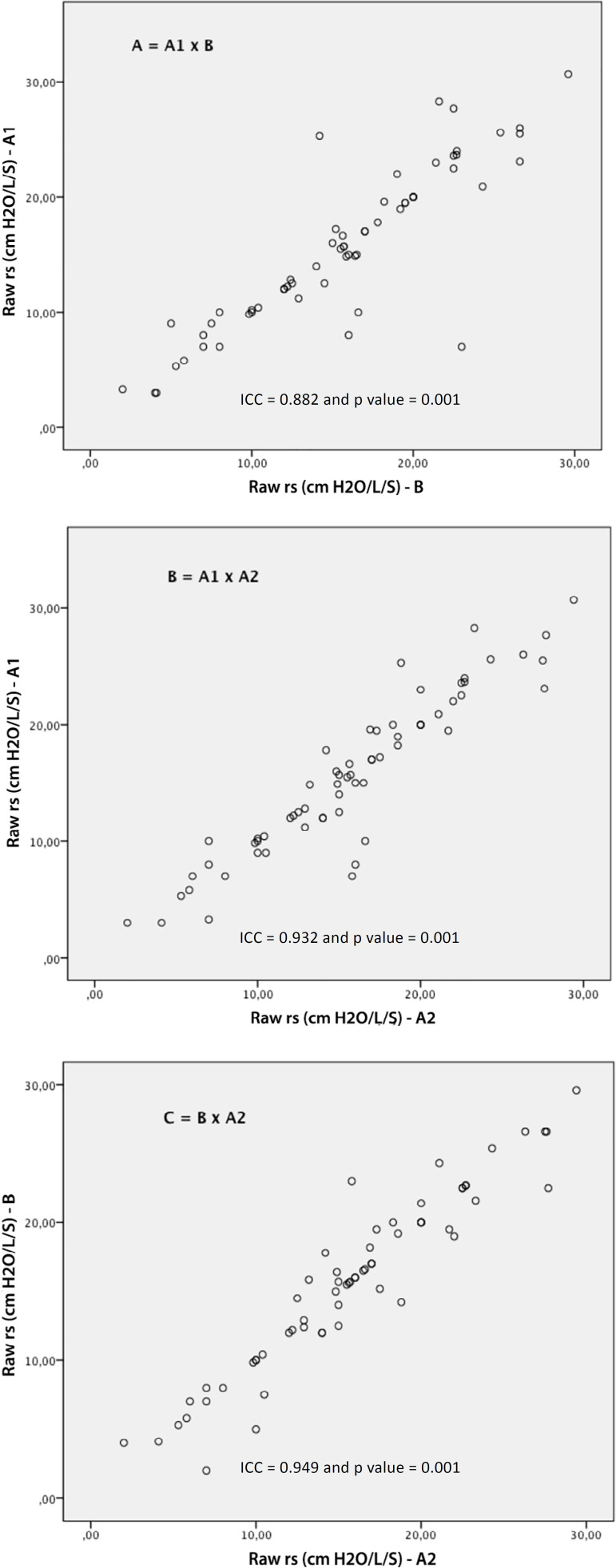
Analysis of the intraclass correlation coefficients for the measurements of respiratory system resistance, interexaminer (A = A1 x B and C = B x A2) and intraexaminer (B = A1 x A2). ICC - intraclass correlation coefficient; Raw - airway resistance.

The following values were found: for Cst rs (A1: 42.1 ± 13.7; A2: 42.2 ± 14.5 and B: 42.4 ± 14.6mL/cmH_2_O); Cdyn rs (A1: 21.3 ± 7.3; A2: 21.3 ± 6.2 and B: 21.4 ± 7.5mL/cmH_2_O); and Raw (A1: 15.7 ± 6.8; A2: 15.9 ± 6.2 and B: 15.7 ± 6.4cmH_2_O/L/s). Excellent reproducibility was observed in the ICC analysis, as shown in figures 2 to 4. The following ICC values were found: for Raw, interexaminer (A1 x B: R = 0.882 and p = 0.001); (B x A2: R = 0.949 and p = 0.001), and for Raw, intraexaminer (A1 x A2: R = 0.932 and p = 0.001); for Cst rs, interexaminer (A1 x B: R = 0.951 and p = 0.001) and (B x A2: R = 0.958 and p = 0.001) and for Cst rs, intraexaminer (A1 x A2: R = 0.965 and p = 0.001); for Cdyn rs, interexaminer (A1 x B: R = 0.957 and p = 0.001); (B x A2: R = 0.946 and p = 0.001) and for Cdyn, intraexaminer (A1 *x* A2: R = 0.926 and p = 0.001).

## DISCUSSION

The present study showed good intra- and interexaminer reproducibility and accuracy for measurements of pulmonary mechanics in mechanically ventilated patients, which reinforces the reliability of this measurement in daily clinical practice for the diagnosis and longitudinal monitoring of changes in the impedance of the respiratory system.

An important aspect of the study is that there was no significant difference, from a clinical standpoint, among the three measurements of respiratory mechanics for Cst rs, Cdyn rs and Raw, which may support the need for only one measurement rather than three, as commonly performed. This would add value to the care provided by the health team, as it would reduce the time required to perform these measurements.

The methods used to measure respiratory mechanics can be dynamic or static. In dynamic measurement, the flow is not interrupted; that is, the mechanical ventilator’s own algorithm provides the result based on the curves obtained, associating them with the equation of motion.^(10)^ In the present study, static monitoring, the most commonly used form in clinical practice,^(10)^ was used; in static monitoring, the flow is interrupted, and the lung compliance and Raw values are obtained. Another point to be reported is that the pause time required to obtain this flow interruption and measure Cst rs was 0.5 seconds, which differs from some studies that report a pause time of 2.0 seconds.^(7,11)^ The justification the pause time selected for the present study is that 0.5 seconds was sufficient for stabilizing the air in the alveoli to obtain the plateau pressure. The use of inadequate pause times can generate incorrect measurements, and longer times than necessary can expose the patient to greater pulmonary stress.

Daoud et al.^(12)^ evaluated the accuracy of the lung mechanics measurements displayed by the mechanical ventilator using the least squares method, which uses the equation of motion together with pressure, volume and flow data, to estimate lung compliance and resistance. The authors observed that these values are not reliable, especially during active breathing, as they overestimate compliance and underestimate resistance. The use of occlusion at the end of inspiration when performing these measurements was selected because this technique is easy and fast to perform in clinical practice;^(12)^ thus, this technique was used in all measurements performed in the present study to ensure the nonoccurrence of measurement bias.

In the present study, the mean Cst rs values obtained were similar to those found in another study^(6)^ and remained below the values found by others.^(11,13)^ The total respiratory system compliance in ventilated and anesthetized patients is approximately 70 - 80mL/cmH_2_O, which almost double the measurement found in the present study.^(14)^ In turn, Arnal et al. analyzed the respiratory mechanics properties of ventilated patients and observed mean Cst rs values of 54mL/cmH_2_O in subjects without lung disease.^(15)^ Although the clinical importance of such evaluations is well established, care teams face a lack of predictive values for comparison with the value they find, and only longitudinal comparisons of these values is possible. This reinforces the need to develop predictive equations for respiratory mechanics.

Regarding the importance of measuring respiratory mechanics in daily practice, some researchers report that the measurement of static pulmonary compliance is associated with the prognosis of patients on MV in terms of the duration of MV and admission to the ICU.^(16)^ Kock et al. assessed the risk of changes in respiratory mechanics for the determination of outcomes such as mortality and observed that the measurement results are strongly associated with the risk of death.^(6)^ These data reinforce the clinical importance of these measurements for the management of critically ill patients.

The applicability of the static measurement of respiratory mechanics as a strategy for the prevention of MV-induced injury is already well established, especially in patients with acute respiratory distress syndrome.^(17)^ This strategy aims to reduce biotrauma, which involves an inflammatory response generated by the biophysical forces applied to the lung parenchyma and which is associated with hyperdistension and the cyclic opening and closing of the alveoli. Therefore, the use of VT, distension pressure < 15cmH_2_O and the maintenance of a plateau pressure < 30cmH_2_O are recommended.^(1,3,7)^

This was the first study to evaluate the inter- and intraexaminer reproducibility of lung mechanics measurements, and the excellent reliability found through the ICC values (> 0.75) ensured uniformity among evaluations. In addition, the method used to perform the measurement is the most accessible and commonly used method in clinical practice.

One limitation of this study was the need for the patient to be fully sedated to measure the mechanics. This limited a larger sample size because to obtain the most accurate measurement, the patient must not exhibit respiratory muscle effort. This factor also influences the use of respiratory mechanics measurements more routinely in daily practice as patients are increasingly unsedated and participating in MV. In the present study, there was no request for increased sedation, and no alveolar hyperventilation was performed to inhibit the respiratory drive and allow the subsequent measurement of mechanics. Another limitation is that different mechanical ventilators were used, which may have affected the values obtained.

## CONCLUSION

The measurement of respiratory mechanics showed good intra- and interexaminer reproducibility for measurements of the resistance and static and dynamic compliance of the respiratory system in patients on invasive mechanical ventilation.
